# Role of physical and mental training in brain network configuration

**DOI:** 10.3389/fnagi.2015.00117

**Published:** 2015-06-23

**Authors:** Philip P. Foster

**Affiliations:** ^1^Department of Nano Medicine and Biomedical Engineering, The Brown Foundation, Institute of Molecular Medicine for the Prevention of Human Diseases, The University of Texas Health Science Center at Houston - Medical SchoolHouston, TX, USA; ^2^Pulmonary, Sleep and Critical Care Medicine, Department of Internal Medicine, The University of Texas Health Science Center at Houston - Medical SchoolHouston, TX, USA

**Keywords:** Alzheimer's disease, schizophrenia, depression, Yoga-meditation, video games, fluid intelligence, spatial memory, SNPs

## Abstract

It is hypothesized that the topology of brain networks is constructed by connecting nodes which may be continuously remodeled by appropriate training. Efficiency of physical and/or mental training on the brain relies on the flexibility of networks' architecture molded by local remodeling of proteins and synapses of excitatory neurons producing transformations in network topology. Continuous remodeling of proteins of excitatory neurons is fine-tuning the scaling and strength of excitatory synapses up or down via regulation of intra-cellular metabolic and regulatory networks of the genome-transcriptome-proteome interface. Alzheimer's disease is a model of “energy cost-driven small-world network disorder” with dysfunction of high-energy cost wiring as the network global efficiency is impaired by the deposition of an informed agent, the amyloid-*β*, selectively targeting high-degree nodes. In schizophrenia, the interconnectivity and density of rich-club networks are significantly reduced. Training-induced homeostatic synaptogenesis-enhancement, presumably via reconfiguration of brain networks into greater small-worldness, appears essential in learning, memory, and executive functions. A macroscopic cartography of creation-removal of synaptic connections in a *macro-network*, and at the intra-cellular scale, *micro-networks* regulate the physiological mechanisms for the preferential attachment of synapses. The strongest molecular relationship of exercise and functional connectivity was identified for brain-derived neurotrophic factor (BDNF). The allele variant, rs7294919, also shows a powerful relationship with the hippocampal volume. How the brain achieves this unique quest of reconfiguration remains a puzzle. What are the underlying mechanisms of synaptogenesis promoting communications *brain* ↔ *muscle* and *brain* ↔ *brain* in such trainings? What is the respective role of independent mental, physical, or combined-mental-physical trainings? Physical practice seems to be playing an instrumental role in the cognitive enhancement (*brain* ↔ *muscle com*.). However, mental training, meditation or virtual reality (films, games) require only minimal motor activity and cardio-respiratory stimulation. Therefore, other potential paths (*brain* ↔ *brain com*.) molding brain networks are nonetheless essential. Patients with motor neuron disease/injury (e.g., amyotrophic lateral sclerosis, traumatism) also achieve successful cognitive enhancement albeit they may only elicit mental practice.

A long-standing question in the neuroscience of aging has been the interplay between physical activity and mental practices, especially to prevent the potential aging-related cognitive decline in reasoning, executive function, processing speed, memory, and spatial ability. Intelligence spans unequally among individuals and follows approximately a normal distribution in the population (Deary et al., 2010). Converging lines of evidence indicate that intelligence is not confined to a limited brain area. Rather, the “connectome” or structural network configuration of brain regions correlates to individual differences in intelligence (Bullmore and Sporns, 2009; Sporns and Bullmore, 2014). Based on diffusion tensor imaging (DTI), white matter integrity, particularly long-distance white matter association tracts, such as arcuate and uncinate fasciculi, has been correlated to increased cognitive ability (Turken et al., 2008; Deary et al., 2010). Clearly, it seems that intelligence requires both integrity and high degree of organizational efficiency of white matter networks. The existence of rich-club networks and resting-state networks (RSNs) became fundamental in cognitive neuroscience. Modeling the topology of rich-club networks from DTI data has been instrumental to determine the dysfunction of their connectivity in patients with schizophrenia (van den Heuvel et al., 2013). However, the underlying mechanisms of physical and mental training on brain networks still remain, for the most part, elusive. Despite accumulation of growing evidence for brain networks' remodeling, synaptogenesis, and neurogenesis resulting from extensive communications intra- or inter-organs, (*brain* ↔ *brain com.*) and (*brain* ↔ *muscle com.*), during motion, activities, exercise training, exergaming, exercise simulation, spatial memory stimulation, mental training, social interactions, or meditation, the hierarchical relationship of those communications has yet to be clarified.

## Original configuration

**Figure 1:**
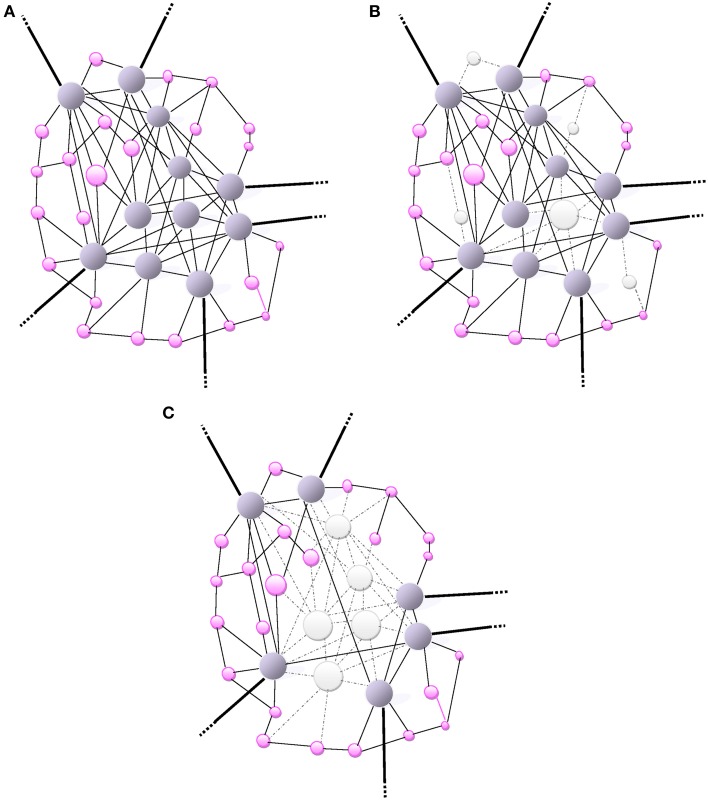
**(A)** Small-world network: A scale-free network. The size of a node (vertex) is proportionally related to the number of connections (edges) that links it to other nodes in the network. Highly-wired nodes with high degree of centrality or high connectivity are illustrated by large blue spheres. Small pink spheres represent low-degree nodes. Hubs (blue spheres), often interact with other regions or “community” modules within the global network; they are highly connected and have high centrality. The bold edges depict connections with other modules or regions. **(B)** The scale-free network (same as in **A**) is inhomogeneous thus resilient as it is relatively minimally affected by a random and non-directed aggression toward a few high-degree nodes mainly causing the loss of more frequent more profuse low-degree nodes. The aggression is visually represented by the loss of nodes (white) and missing edges (dotted lines). **(C)** An informed agent aggressing high degree nodes and hubs. Visually, the network architecture is completely de-structured with the detrimental loss of a major number of connections (loss nodes in white, missing edges as dotted lines).

**Figure 2:**
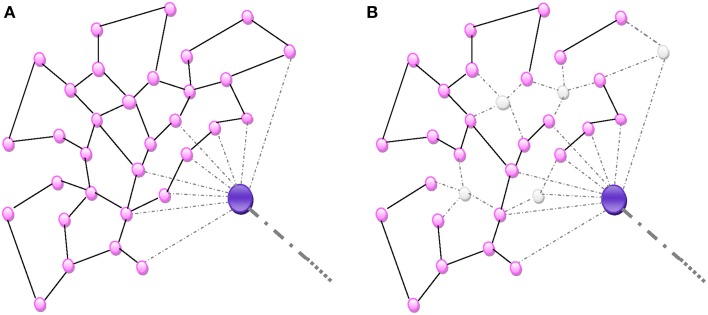
**(A)** Random homogeneous network. All connections are random and equally probable. **(B)** An aggression destroying nodes de-structures the homogenous network proportionally to the number of nodes.

The inference of interventions (physical or mental) on the brain relies on the flexible network architecture and may be viewed from two standpoints, the global aspect of network topology and local remodeling of proteins and synapses of excitatory neurons. It is hypothesized that the topology of brain networks is characterized by a number of connecting nodes in four dimensions (space and time) which may be remodeled throughout a lifetime by appropriate training. The degree of a node is the number of connections (edges) or, more precisely, its probability, *P*(*k*), of interacting with other nodes in the network (Barabasi and Albert, 1999; Albert et al., 2000). Although somehow random, connections are not completely chaotic. Rather, the probability distribution function *P*(*k*) frequently follows a scale-free power-law [*P*(*k*) = *k*^−γ^], with γ usually ranging between two and four (Barabasi and Albert, 1999; Albert et al., 2000). Connections are governed by two underlying mechanisms, growth and preferential linking (Barabasi, 2009). New nodes prefer to link with most connected nodes (Figure 1A), the more “popular” nodes (Barabasi, 2009) so they may then become hubs, with high degree or “high centrality” (Bullmore and Sporns, 2009). It is conceivable that mental and physical training-induced synaptic plasticity promotes greater connectivity, shorter path length, global efficiency (Gard et al., 2014), via amplification of the number of preferential linking. Elected networks would then become more clustered. However, brain networks configuration may occasionally develop more degree of built-in randomness as they would follow a trend toward a Poisson distribution (Figure 2) with an exponentially truncated power law, suggesting that the probability of highly connected (“high-degree”) nodes (hubs) is greater than in an equivalent random network, but less than would be anticipated in a scale-free network (Barabasi and Albert, 1999; Bullmore and Sporns, 2009). From small size to large-scale, the brain network architecture configures itself into a scale-free stationary state with a fixed distribution [*P*(*k*)] which is also independent of time (Barabasi and Albert, 1999). In such a small-world model, *n* nodes form a one-dimensional lattice, each node being linked to its two nearest, next-nearest neighbors and so forth (Albert et al., 2000; Barabasi and Albert, 1999). With probability *P*(*k*), each node is connected to another rather randomly selected node. The long-distance connections produced by this configuration decrease the distance between nodes, leading to a small-world property with an average of approximately “six degrees of separation” (Barabasi and Albert, 1999). Human functional brain networks ubiquitously express small-world properties with high clustering, high global efficiency, aggregation in “community” modules, path length minimization, and highly connected nodes (hubs) (Bullmore and Sporns, 2009, 2012). Those highly-clustered large-scale brain networks are anatomically close and functionally associated. A recent study highlighted the greater small-worldness resulting from mental and physical training (Gard et al., 2014). Some regions, RSNs, expressing consistent signal variations resulting in high internal functional connectivity have been recently identified by intrinsic or resting-state functional magnetic resonance imaging (fMRI) (van den Heuvel and Sporns, 2011; van den Heuvel et al., 2013; Sporns, 2014).

## Aging and network resilience

The dysfunction or loss of any node increases the distance between the remaining nodes, as it can eliminate some paths that contribute to the small-world connectivity. The inter-nodal distance remains unchanged under an increasing number of errors. Therefore, a small-world connectivity between the remaining nodes in the network would not be altered albeit over 5% of the nodes had failed (Albert et al., 2000). The resilience of scale-free networks relies on their remarkably heterogeneous connectivity distribution. Because the network configuration is regulated by the power law, the probability of failures in a very low number of highly connected nodes is extremely small compared with the more likely occurrence of failures or losses in a multitude of poorly connected nodes (Albert et al., 2000). The architectural path of remaining nodes is unaffected by the failure of those poorly-connected non-essential nodes so that the global network topology would subsist almost unaltered over time (Figure 1B). However, during the aging process this small-world resilience may be impaired by the overwhelming random number of failures leading to a greater probability of affecting high-degree nodes. In addition, the repair process of failed nodes may be impaired with aging. Therefore, the functional integration becomes reduced by lengthening of the characteristic path length of networks.

## Instantaneous brain network response to exercise

A hint on how patients with neuron disease/injury (e.g., amyotrophic lateral sclerosis, traumatism) may stimulate specific exercise-sensitive brain networks without any motor activity is provided by the following experiment. Hypnotic suggestion of exercising “uphill” elicits an increased breathing response (rate and minute ventilation, $\dot{V}$_E_) (Paterson, 2014). If the respiratory muscles are not too severely affected by the neuro-muscular disease such patients may respond to the suggestion and possibly to autosuggestion. The terminology “central command” has been used to express the physiological modulation of cardiorespiratory response to a constant exercise or a simulation of exercise (Paterson, 2014). Imaginary exercise (imagining cycling uphill) induced activation of the dorsal lateral prefrontal cortex (DLPFC), superolateral sensorimotor cortex (SLSMC), lateral sensorimotor cortex (LSMC), and supplementary motor area (SMA) as seen by positron emission scanning (Paterson, 2014). Voluntarily breathing alone produced solely an activation of sensorimotor cortex (SLSMC, LSMC) and SMA. A remarkable finding was that only imaginary exercise was amenable to produce the activation of the DLPFC which is an important area for executive function and working memory. Projection tracts are identified from the DLPFC to the SMA, premotor area and to the lateral periaqueductal gray (LPAG). The periaqueductal gray (PAG) has also been shown to be an important component of the brain network regulating the “central command.” The LPAG also sends abundant projections to the hypothalamus: the effects on cardio respiratory parameters are reflected by an increase in heart rate, mean systemic arterial pressure and respiratory rate during the anticipation phase of exercise. It has been suggested that the PAG serves as a “cognitive integrator” which also receives input from the medial prefrontal cortex, amygdala, hypothalamus (Paterson, 2014). There is a plethora of information circulating in brain networks during exercise albeit a question remains: how the extremely plastic dentate gyrus (DG) would be connected in those brain networks activated during exercise? How spatial recognition and landmarks would be involved in this system of activation?

## Energy cost of synaptogenesis and network failure

The energy cost of a node is proportional to its degree, its centrality (hub), and to the length of the inter-nodal connection (Bullmore and Sporns, 2012). The centrality and connectivity of nodes also depend on the ability of neurons to support the synaptogenesis in terms of metabolic requirements. Mitochondria are concentrated in subcellular area of high metabolic prerequisite for neuronal growth (active growth cones) or plasticity and increased density in axon presynaptic terminals (Li et al., 2004). At the sub-cellular level, cellular growth is also controlled by synergy of intra-cellular metabolic and regulatory networks for the genome-transcriptome-proteome interface (Maslov et al., 2009). A measure of synaptogenesis may be achieved by looking at the accumulation of postsynaptic NMDA (*N*-methyl-D-aspartate) receptors. The timescale for synaptogenesis appears to occur rather rapidly between tens of minutes to possibly 1–2 h. However, a dysfunction of high-energy cost wiring such as hubs may occur, producing long-range connections and brain network abnormalities which are a hallmark of Alzheimer's disease (AD).

Clearly, Alzheimer's disease is a model of energy cost-driven small-world network disorder. The number of long-range connections is reduced, increased clustering and the path-length is greater as the network global efficiency is impaired (Bullmore and Sporns, 2012). Such a pattern of small-world network reconfiguration may be considered as a shift in the direction of lowering energy connection cost albeit allowing the sacrifice of data integration ability. Small-world networks consistently identified as high-degree hubs, such as the medial posterior parietal cortex, are also the first brain areas where deposits of amyloid-*β* (A*β*) levels proteins aggregate (Figure 1C). In the case of (A*β*) selective deposition and damage to high-energy network connector hubs, a disproportionate detrimental impact on the global efficiency of small-world networks is anticipated (Albert et al., 2000). The natural resilience of scale-free network to an aggression may be overwhelmed by an informed agent, i.e., (A*β*) deposition, selectively targeting high–degree nodes (hubs) as illustrated in Figure 1C. When most highly connected nodes are damaged, the diameter of the scale-free network augments rapidly and loses efficiency (Albert et al., 2000). The loss of most hubs leads to an alteration of the small-world network topology and the capacity of the remaining nodes to communicate is highly impaired. The vulnerability of brain small-world networks to non-random aggression by informed agents is inherent to the heterogeneity of the connectivity distribution. The property of resilience deep-rooted in the heterogeneity of the connectivity becomes powerless when facing a selective targeting of highly connected nodes in the small-world.

## Fine-tuning the strength of excitatory synapses and remodeling errors

Homeostatic neuronal plasticity via physiological synaptic scaling is essential for stabilizing neuronal network function. Overall remodeling of proteins of excitatory neurons is fine-tuning the scaling and strength of excitatory synapses up or down via activation of AMPA receptors, BDNF, guanylate kinase–associated protein (GKAP), TNF-*α* and all-trans retinoic acid (Shin et al., 2012). On a macroscopic scale of four dimensions, the degrees of nodes are modified and the small-world networks' architectures are continuously remodeled into new configurations. External treatment such as physical or mental training may also infer with the homeostatic scaling and reconfigure brain networks.

**Figure 3:**
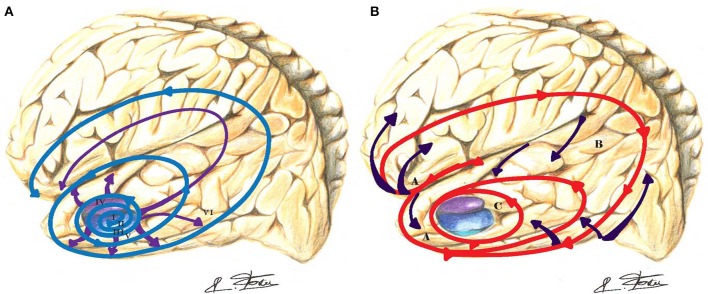
**(A)** In Alzheimer's disease (AD), the outward progression of neurofibrillary lesions spreads from the entorhinal cortex toward the cortex (blue-purple spirals and arrows). Those lesions are crossing and blocking the path of long-distance white matter tracts. Braak stages are in Roman numerals. **(B)** The inward cortical myelination maturation of long-range white matter tracts is directed toward the entorhinal cortex (red spiral and purple arrows) and replicates in reversed order the neurofibrillary lesions of AD.

In contrast, hippocampal long-term potentiation (synaptic plasticity) underlying information storage, involved in learning and memory, is inhibited by (A*β*) deposits (Jo et al., 2011). An increased caspase-3 activity via the mitochondrial pathway of apoptosis has been observed in spines of transgenic mouse models of AD (Erturk et al., 2014). This process may lead to a loss of spines and synapses and abnormal excessive pruning in AD. Visualization (ultra-high field 7-T MRI) of the hippocampal CA1 apical neuropil layer thinning in subjects with mild AD suggests a greater role for synaptic loss than neuronal loss (Kerchner et al., 2010). The typical AD (Dubois et al., 2010) involves initial intra-neuronal neurofibrillary lesions of the entorhinal cortex, the hippocampus, and related medial temporal structures, which subsequently diffuses to the iso-cortex and neocortical association areas (Braak and Braak, 1991). Figure 3A depicts the progression and chronology of the spatial deposition of (A*β*) in the brain. The characteristic outward progression of neurofibrillary lesions (Figure 3A) from the entorhinal cortex mirrors in reversed order the inward cortical myelination maturation of long-range white matter tracts (Braak and Braak, 1996) (Figure 3B). The spreading of these lesions is precisely crossing and disrupting the crucial paths of long-range white matter tracts. Indeed, lesions of long-range white matter tracts may produce weaker or dysfunctional connectivity in those AD patients (Grady, 2012).

## Cartography of evolving and growing brain networks: general concept

Creation-expansion of synapses in constantly evolving brain networks may be described in an *n-dimensional Cartesian space* (*n*-*space*). The above macroscopic description of creation-expansion-removal of synaptic connections in a *macro-network* takes place in a four-dimensional space. Heterogeneity exists within *macro-networks*, with a first level of fractal scaling reflecting the number of projections to or from an area of the brain: regions of gray matter with high density of nodes and more projections in accordance with a power law (Bullmore and Sporns, 2012). The fractal or Rentian scaling exponent of the human brain network serves to evaluate the allometric scaling relationship between gray-matter volume and white-matter volume over a range of species (Bullmore and Sporns, 2012). Other *fractal dimensions*, at the sub-level of intra-cellular scale, via *micro-networks* or *interactome-networks* regulate the physiological mechanisms of the selection process in determining the preferential attachment of synapses and will affect by feedback the cartography of brain *macro-networks*. The idea of such micro and macro-scale as a model of the human brain has already been commented (Sporns, 2013) and will be elaborated upon in the next two sections.

## Brain “macro-networks”

Expansion and growth of brain networks, by continuous addition of new vertices or nodes (e.g., synapses–neurons) and links, or edges (e.g., axons–dendrites), between nodes, reflects both a random and deterministic process which can be mathematically described in a *4-D* space. New attachments probability depends on the criticality of targeted synapses in a network, e.g.,*de novo* creation from arising dendritic spine synapse or pre-existing and potentially expanding synapse. As aforementioned, the probability distribution function (PDF) follows a power law. Brain networks may be viewed as structural (anatomical) wiring cartography or as functional mapping of neuronal activity and individuation of nodes is rather straightforward in neuronal networks (Sporns, 2014). Connections weights based on myelination degree may also be evaluated by DTI albeit the individuation of nodes is limited by methodological issues since partitioning limits are not superimposed with connectivity mapping and may result in the clouding of networks connections (Sporns, 2014). These techniques have led to the identification of network communities or modules such as RSNs with coherent signal fluctuations producing high internal functional connectivity (Vincent et al., 2007). Existence of brain networks with high centrality and high interconnectivity has then been characterized by DTI, using streamline tractography with individual parcellation map, and fMRI (van den Heuvel and Sporns, 2011, 2013; van den Heuvel et al., 2013). Hub region with high degree are prone to be highly mutually connected to each other and form a sub-network (“rich-club”) but more reciprocally connected than previously hypothesized for degrees with high degree properties (van den Heuvel and Sporns, 2011; van den Heuvel et al., 2013). The rich-club is playing a major role in brain's network topology (Goni et al., 2014). In patients with schizophrenia, the interconnectivity and density of rich-club networks are significantly reduced in white matter projection tracts that link midline frontal, parietal, and insular hub regions (van den Heuvel et al., 2013). In those patients, the selective disruption of brain connectivity among rich-club (frontal and parietal hubs) regions of the brain is associated with an impairment of global communication capacity, an impairment which is lacking in alterations of other white matter pathways (van den Heuvel et al., 2013). Another observation in those patients with schizophrenia was an increased coupling between structural and functional connectivity (van den Heuvel et al., 2013).

### Attempts to quantify the general process of macro-remodeling

The “*preferential attachment*” hypothesis postulates that the rate Π(*k*) at which a node, with initial *k*-links, gains new links is a monotonically increasing function of *k* (Jeong et al., 2003). The larger is *k*, the greater affinity for attracting new linkages by this “popular” node. It has been proposed that the *k*-links' distribution follows a power law (Barabasi and Albert, 1999; Albert et al., 2000). As a result, the time course of the *k*-links' distribution, or degree *k_i_*of the *i*^th^ node may be obtained by the differential equation (Jeong et al., 2003)

(1) $$\frac{\text{d}k_{i}}{\text{d}t}=m\Pi {\left(k\right)}_{i},$$

where *m* is a constant, and Π(*k*) may be expressed as follows

(2) $$\Pi {\left(k\right)}_{i}=\frac{k_{i}^{\gamma }}{\sum_{j}k_{j}^{\gamma }},$$

with γ > 0 an unknown scaling exponent. When γ = 1, Equation (2) becomes a scale-free model (Barabasi and Albert, 1999), and the probability distribution function (PDF), *P*(*k*), frequently follows a scale-free power-law [*P*(*k*) = *k*^−^^γ^], with usually γ = 3. As the network expands, the function Π(*k_i_*) provides the rate of acquisition of new links by a pre-existing node with *k*-links. Knowing Π(*k*) requires evaluation of how old nodes acquire new nodes, as a function of the degree of the old node.

**Figure 4:**
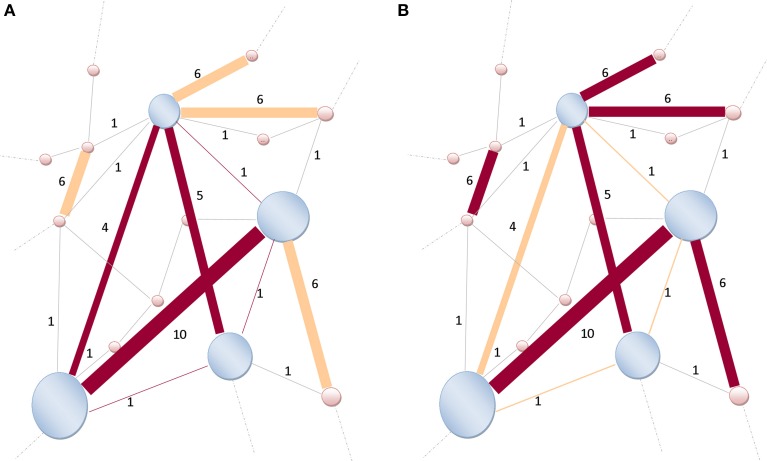
**Illustration of a rich-club network. (A)** The size of nodes is proportional to their richness. High-degree nodes are shaded-blue, lower-degree are shaded-pink. The weights (strength) assigned to the links are indicated by Indo-Arabic numerals. Links (dark brown) are connecting nodes (shaded-blue) within the rich-club network. Only six links are connecting those high-degree nodes together, thus *k*_>_*r*__ = 6, and *W*_>_*r*__ = 4 + 10 + 5 + 1 + 1 + 1 = 22. **(B)** Not all strongest *k*_>_*r*__ = 6-links (dark brown) of the entire network are connecting rich-club network's nodes (shaded-blue). Some strong links are also connecting lower-degree nodes (shaded-pink). Therefore, the denominator of Equation (3) becomes $\sum_{l=1}^{k_{>r}}w_{l}=6+10+5+6+6+6=39$ and the ratio is readily obtained as Π(r)=22/39. Modified and re-sampled from Opsahl et al. (2008).

The following section describing mathematical modeling of rich-club networks has been used in the analysis of data (DTI) from patients with schizophrenia to identify the dysfunction of connectivity of those rich-club networks (van den Heuvel et al., 2013). The tendency to engage in stronger and preferential interaction is called the weighted rich-club effect. A way to formalize this idea is to classify nodes by their degree (popularity) (Opsahl et al., 2008). Nodes with high degrees rising to preponderance in the network are those through which most of the information within the network is transiting. All nodes in a network may be ranked by a richness parameter, *r* (Opsahl et al., 2008). At each value of *r*, the set of all nodes with richness larger than *r* is selected (the rich-club). A series of gradually upscale rich-clubs is then selected. For each rich-club, the number of links (*weak and strong*), *k*_>_*r*__, connecting nodes is counted, and the sum of weights, *W*_>_*r*__, assigned to these links, measured (Opsahl et al., 2008). The fraction of weights assigned to links shared by the “rich nodes” divided (compared) to the total amount (sum) of weights that could have been shared, should they had been connected via the *strongest k*_>_*r*__ − links in the network (Opsahl et al., 2008) becomes

(3) $$\Pi (r)=\frac{W_{>r}}{\sum_{l=1}^{k_{>r}}w_{l}},$$

where the ranks *w_l_* ≥ *w*_*l*+1_, with *l* = 1, 2, …, *k*, are the assigned weights on the links belonging to the rich-club network, and k is the total number of links (Figure 4). The only caveat of Equation (3) is the possibility of having a non-zero value for Π(*r*) in the case of links fully randomly selected. To avoid this and remain comparable to real networks, the introduction of a null model discounting the random assignment of links preserves the PDF of various variables: *P*(*k*) [probability that a given node is connected to *k* neighbors]; *P*(*w*) [probability that a given link has a weight *w*]; and *P*(*r*) [probability that a given node has richness *r*] (Opsahl et al., 2008). At this phase, a series of re-shuffling processes for a variable take place while keeping intact the other variables, e.g., re-shuffling weights globally in the network while topology is invariant (Opsahl et al., 2008). For a given assignment of richness, *r*, the weighted rich-club effect can be evaluated by the ratio

(4) $$\Phi (r)=\frac{\Pi (r)}{\Pi_{null}(r)},$$

where Π_*null*_(*r*) is an estimate of the rich-club effect (Opsahl et al., 2008). When Φ(*r*) is greater than 1, the network of interest has a positive weighted rich-club effect. To the contrary, if Φ(*r*), is smaller than 1, links in the network of interest are weaker than randomly predicted.

### Macro-remodeling as a function of time

Another way to express Equation (1) is to observe the addition of new synapses (nodes) in a small time-interval Δ*t*, starting at time *t*, and ending at *t* + Δ*t*. Thereby, instead of Π(*k*), it is also possible to study *F*(*k*), the cumulative distribution function (CDF) of new nodes (e.g., synapses) creation, defined as (Jeong et al., 2003)

(5) $$F(k)=\int_{0}^{k}\Pi (k_{i})dk.$$

The same treatment may be applied to the variation of the rich-club effect in time by studying Φ(*r*) (Equation 4) so that the CDF of the rich-club effect becomes

(6) $$F(r)=\int_{0}^{r}\Phi (r)dr.$$

However, the overarching question is not whether the CDF,Π(*k*), of the overall growth of the brain network follows a fixed function. Such a function ignores the elementary processes, essential components, in the selection process of creating, expanding or removing nodes in a constantly evolving real network (Ghoshal et al., 2013). Rather, how elementary processes underlying preferential attachments, e.g., related to the aging-fitness of nodes, availability-functionality of genomic and proteomic intra-cellular systems, and preferential paths of action potential would regulate the network evolution?

### Decision, at the elementary level, of macro-remodeling

**Figure 5:**
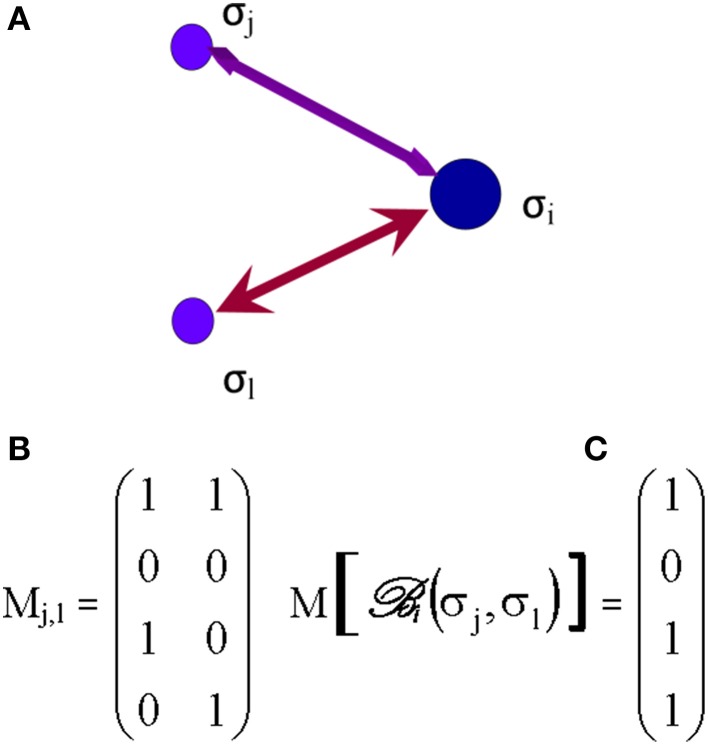
**The Boolean matrix (C) defines ${ℬ}_{i}$, or adjacency matrix (in graph theory) whose rows and columns** (***right matrix M*** **[**${ℬ}_{i}\left(\sigma_{j},\sigma_{l}\right)$*], C*) **represent the nodes (e.g., synapses) and whose entries represent the edges (e.g., axons) of the graph** ***(left matrix M_j,l_, B**)*. The attribution of the output ${ℬ}_{i}\left(\sigma_{j},\sigma_{l}\right)$ = σ_*i*_, e.g., to Synapse *i*, to each input $\left(\sigma_{j}\right)$ and $\left(\sigma_{l}\right)$ depends on the values of those inputs (0 or 1) and the order of first edge activation/stimulation $\left(\sigma_{j},\sigma_{l}\right)$ or $\left(\sigma_{l},\sigma_{j}\right)$ (initial propagation of an axonal action potential) as illustrated on **(A)**. The values of ${ℬ}_{i}\left(\sigma_{j},\sigma_{l}\right)$ have been arbitrarily selected for the example shown.

Based on several inputs from those elementary processes, received at time *t*, each *i*^th^ synapse, inasmuch as one or more synapses may represent one node (or vertex), makes its own decision about its state and the selection of future connections. A way to assess the selective process by which a synapse follows its own agenda is the introduction of Boolean networks (Albert and Barabasi, 2000) in the next phase of the decision tree. In brain networks, the Boolean model would be made up of *N* synapses (vertices) which are defined by Boolean variables (spins) that may take values σ_*i*_ = 0 (not connected) or 1 (connected). Each synapse may receive inputs from *k* synapses. Potential random values of the spins selected from these neighboring synapses are as much as *2^k^*. The Boolean function, ${ℬ}_{i}$, grants each of these *2^k^* inputs (Albert and Barabasi, 2000), an output value of 1 (e.g., synaptic connection established) or 0 (no further durable connection). The outputs are randomly selected, assigning a value 1 for a probability *P* (will be connected) and a value 0 for a probability (1–*P*) (will not be connected). The Boolean matrix determines, at any time *t*, the spin of the *i*^th^ synapse, based on the spin of its *k* neighboring synapses. Figure 5 illustrates the case of *k* = 2, where Node *i* may receive four potentially different inputs from two neighboring synapses, *j* and *l* (Figure 5A).

## Intra-cellular “micro-networks”

In a fractal dimension of lower scale, micro-networks exhibit similar patterns as macro-networks. However, different assignments are specific to the micro-scale so that vertices may become macromolecules, while the edges are biophysical, biochemical, and functional interactions of DNA (gene sequences), RNA or metabolites (Vidal et al., 2011). In a transcriptional regulatory network, nodes correspond to transcription factors or presumed DNA regulatory elements, and edges represent the chemical binding between the two. In systems biology, four types of micro-networks are used to describe biological processes (Le Novere, 2015).

The *interactome-networks* are generally used to evaluate whether *X* affects *Y* (physical or chemical interaction), e.g., between genes and proteins. The ENIGMA Consortium looked at the intergenic single nucleotide polymorphisms (SNPs) influencing the hippocampal volume (Stein et al., 2012). In this particular example, one SNP shows a powerful relationship with the hippocampal volume normalized to intracranial volume. The SNP, locus rs7294919, of gene *RPL 6P15*, on chromosome 12, was associated with hippocampal volume. In this case, *X* (allele variant rs7294919) is known, the protein *Y* not fully identified, and the phenotype, at macro-network fractal scale, is the hippocampal volume. This SNP, rs7294919, was presumed to regulate the transcriptional activity of a neighboring gene, *TESC*, and its end-product tescalcin (Stein et al., 2012; Dannlowski et al., 2015). Tescalcin (*Y*?) may enhance hippocampal cell proliferation and differentiation.

The *activity flow networks* provide additional information about whether *Y* is increased or decreased. As an example, most synaptic genes (*X*) are upregulated in mild cognitive impairment (MCI) compared to age-matched controls, particularly in the hippocampus, superior frontal gyrus, and post central gyrus while being downregulated in AD (Cribbs et al., 2012; Berchtold et al., 2014). The genes *X* may be involved in the encoding and increased production of some proteins (*Y*) such as neurexins, integrins, or cadherins (Berchtold et al., 2014).

The *process description networks* detail and quantify how *X* affects *Y* (directed, sequential, mechanistic); they may be used to describe a mass transfer. An example is provided by a SNP of the *Neuregulin-1 (NRG-1)* gene (*X*); an imbalance in Neuregulin-1 (NRG-1*β*) may disrupt the dopaminergic and glutamatergic functions, neurotransmitter pathways associated with schizophrenia (Kwon et al., 2008). The NRG-1*β* protein (*Y*) exerts its effects on synaptic plasticity within minutes and increase levels of extracellular dopamine levels dopamine in the hippocampus and striatum.

The *entity relationship networks* introduce the directionality of the relationship, e.g., *X* stimulates *Y*, but *Y* does not influence *X*; such networks are used for mapping the cell cycle or apoptosis (Le Novere, 2015). Those micro-networks usually express a scale-free topology and high clustering (Barabasi and Oltvai, 2004). In cells, scale-free networks are ultra-small and their path lengths are short. A short path length implies that a local perturbation of the concentration, [*X*], could affect *Y, Z* and rapidly spread to the entire network—path of two or three reactions away.

Hierarchical or fractal modularity of network topology exists for reconfiguration of connections between nodes (Bullmore and Sporns, 2012). This hierarchical modularity and *macro-networks* homeostasis rely on the optimal functionality of an intact genomic and proteomic intra-cellular system within *micro-networks*. In contrast, the opposite is not true and optimal operation of *macro-networks* does not seem necessary for proper *micro-networks* functionality. However, a hierarchy seems to exist, with a sovereignty of the *macro-networks* over *micro-networks*. In the synaptic *macro-network*, the inference by repetitions of propagation of action potential (e.g., training), on the aging–fitness of nodes (synapses) seems critical to increase the synaptic density.

## Synaptogenesis induction and brain networks

From a structural standpoint, brain plasticity entails the potential of neurons to change their synaptic connections (Ashford and Jarvik, 1985). The lengthening of axons, sprouting of collateral ramifications, and remodeling allow the dwelling of new synapses, new cognitive, and behavioral operations (Foster et al., 2011). Skeletal muscle exercise affects brain plasticity and may arrest, slow down or even reverse the pathophysiological evolution to MCI (Foster et al., 2011).

Aging affects three high-degree and large-scale brain networks modulating interconnectivity between the frontal cortex and the rest of the brain: (1) Default mode network (DMN) (posterior cingulate, ventral–superior frontal medial cortices, and bilateral lateral occipital, middle frontal, hippocampal, and para-hippocampal, and middle temporal cortices]; (2) Fronto-executive network (FE); and (3) Fronto-parietal (FP) network (Voss et al., 2010). Skeletal muscle activity mitigates the age-related deterioration of those networks depending on the quality and length of training. One year of walking increased functional connectivity within the DMN and the FE Networks but non-significant at 6 months (Voss et al., 2010). A group, training in non-aerobic stretching, toning and balance, also showed increased functional connectivity in the DMN after 6 months and in the FP Network after 12 months. It appears that an exercise-induced restoration of age-deteriorated brain networks took place. Improved functional connectivity was associated with greater improvement in executive function and behavioral normalization.

Activity-induced plasticity of spines and synapses models the nodes and the edges of small-world networks. Pruning of synapse exuberance or redundancy to shorter the path length and global efficiency is also an aspect of the normal plasticity of the brain. Healthy brains, especially in the hippocampus, are the sites of continual effervescence of molding new synapses or eliminating others (Sheng and Kim, 2011; Shin et al., 2012; Sporns, 2013). However, pruning may exceed the physiological homeostatic level. In AD, excess of synapse scaling has emerged as an important factor of small-world network disorder (Erturk et al., 2014). Degradation of spatial memory is also an inaugurating sign of cognitive impairment and a potential presentation of AD.

## Exercise and spatial memory activation: simultaneously or isolated?

Training-dependent (physical activity or mental stimulation) reconfiguration of brain networks is essential in learning, memory, and executive functions. Besides exercise, mental practice is known to mitigate the age-related cognitive decline, especially via regular exposures to virtual reality (Anguera et al., 2013; Robert et al., 2014). How the brain achieves this unique quest remains a puzzle. Film directors and video game designers are immersing the audience in their virtual world by causing a widespread brain arousal (Hasson et al., 2004; Hasson and Malach, 2006). Virtual reality requires only minimal motor activity and cardio-respiratory stimulation. Perception of the surrounding environment or virtual world of a computer screen elicits spatial memory in order to localize the characteristic configuration of the scene on display. Perception, motion or navigation in a three-dimensional space are closely related to spatial memory requiring permanent visual tracking of the direction and distance from reference points, or landmarks, and their integration by hippocampal and entorhinal network mechanisms underlying grid cells executive mapping (Hafting et al., 2005).

Skeletal muscle activity during childhood may produce greater adult-like recruitment of anterior prefrontal brain regions which are critical for maintenance and goal-oriented cognitive control (Chaddock-Heyman et al., 2013). Regularly exercise-trained children may learn to better maintain a sustained task that demands selective attention and distraction suppression, reflecting a more mature brain function with a post-training reduction of the anterior prefrontal cortex activation (Chaddock-Heyman et al., 2013). Exercise may also allow protection from the negative cognitive and emotional consequences of inevitable stress (van Praag et al., 2014). There is a growing body of evidence that aerobic exercise training in older humans increases (serum) BDNF levels, and selectively increased the volume of the anterior hippocampus including the DG, where neurogenesis is prone to arise, as well as subiculum and CA1 subfields known to encoding spatial memory (Erickson et al., 2011; Voss et al., 2013b). In humans, an age-related reduction of the number of hippocampal neurons has been observed albeit the DG seems less affected by the loss of neurons (Spalding et al., 2013). The forming of memory representation and recognition (pattern separation) may originate from the DG, via recurrent axons mossy terminals, within CA3, which might be “detonators” for their downstream neuronal targets in the CA3 network (Aimone et al., 2011). Furthermore, DG neurogenesis relies on signals from two separate neuronal populations (Aimone et al., 2011, 2014): (a) “hyperexcitable” immature neurons, incompletely tuned neurons, with yet low connectivity, open to inputs; and (b) sharply tuned neurons. Exercise increases the proliferation of neuroprogenitor cells in the DG (Van et al., 1999, 2005; Deng et al., 2010).

It appears that direct simultaneous stimulation of brain and muscle enhances the communication processes (*brain* ↔ *muscle com*.) and is necessary to produce a superior effect on cognitive benefits. Isolating the effects of the direct stimulation of the brain and muscle by trying to offset the transmission of information between them provides insights into the relevance of the communication process (*brain* ↔ *muscle com*.). Animal research has shown that enriched environment alone does not increase hippocampal neurogenesis but running alone does; skeletal muscle exercise, rather than cognitive stimulation, is required for hippocampal neurogenesis (Mustroph et al., 2012; Voss et al., 2013b; Kobilo et al., 2014).

A study (Anderson-Hanley et al., 2012) brought about further insights into the role of virtual reality-enhanced sub-maximal skeletal muscle exercise training (3 months, 45 min/5 times/week at 60% heart rate reserve). Observing a *3-D* virtual navigation on a computer screen while exercising on a stationary bicycle (“exergaming”) provided greater cognitive (executive) benefits than stationary bicycle alone. Therefore, another mechanism not directly related to muscle contractions and cardiorespiratory stimulation may be responsible for the improvement in cognition.

## Molecular basis for induction of brain network remodeling

### By skeletal muscle exercise

Neurotrophins are essential proteins for neurogenesis. The physiology of neurotrophins may be studied in a systematic way by first looking at their direct effect on the brain and then examining the communication (*brain* ↔ *muscle com*.).

At the brain level, exercise increases BDNF levels in the hippocampus of young and aged brains (Neeper et al., 1995, 1996; Berchtold et al., 2002). Specifically, BDNF mRNA levels were elevated in the DG of running animals (Farmer et al., 2004) rather than in area CA1 (Voss et al., 2013b). The greatest effects of exercise on BDNF seem to target highly plastic, or transformable areas, responsive to environmental stimuli (Volkmar and Greenough, 1972; Castren et al., 1992). The enhancement of neurogenesis and learning in exercising animals may be increased via levels of BDNF (Berchtold et al., 2010; Adlard et al., 2011). Tropomyosin receptor kinase B (TrkB) is a receptor for BDNF, and their post-exercise training levels (7-days regimen) are elevated in the hippocampus of rats (Ding et al., 2011; Voss et al., 2013b). Synthesis of the mature form of BDNF (mBDNF) is produced from the proteolytic cleavage of a precursor protein, proBDNF (Ding et al., 2011). Serum protease tissue-type plasminogen activator (tPA), ubiquitous in the CNS (present in hippocampus), converts plasminogen onto plasmin which in turns cleaves proBDNF (Ding et al., 2011). mBDNF is a major synaptogenesis inductor, acting on intracellular signaling *micro-networks* also involved in synaptic transmission (Ding et al., 2011). BDNF also regulates multiple neurotransmitters, including the dopaminergic, cholinergic, and GABAergic (gamma-aminobutyric acid) systems (Knusel et al., 1991). Evidence for a correlation of serum BDNF, insulin-like growth factor (IGF-1), and vascular endothelial growth factor (VEGF) with an increased functional brain networks' connectivity in the medial and lateral temporal cortices has been demonstrated (Voss et al., 2013a). The strongest relationship with functional connectivity was identified for BDNF and, BDNF activity may be modulated by IGF-1 and VEGF, both inducing the growth of endothelial cells, via expressing nitric oxide synthase (*e*NOS), also required for exercise-induced up-regulation of BDNF in the hippocampus (Voss et al., 2013a). Exercise-induced up-regulation of BDNF, IGF-1, and VEGF may neutralize some age-related diminution of neurogenesis, synaptogenesis in DMN, FP, and FE networks. A growing line of evidence for underlying exercise-induced neurogenesis in the DMN network is also inferred by IGF-1 being a key player in the prevention or annulment of aging-induced cognitive impairment or AD (Carro et al., 2005, 2006).

A way to understand the communication (*brain* ↔ *muscle com*.) is to study peripheral physiological molecular mechanisms. Brain plasticity and spatial memory performance were enhanced by intra-peritoneal administration of AMP-activated protein kinase (AMPK) in wild-type mice (Kobilo et al., 2014). Mice with muscle-specific mutated AMPK *α*2-subunit (AMPK-DN) did not modify their behavior, further providing support for a muscle-mediated mechanism in a (*brain* ↔ *muscle com*.) (Kobilo et al., 2014). In skeletal muscle, the exercise-induced production of peroxisome proliferator-activated receptor-gamma co-activator (PGC-1*α*1) modifies the tryptophan-kynurenine metabolism and protects from stress-induced depression (Agudelo et al., 2014; Moon and van Praag, 2014). This mechanism is mediated by the actions of PGC-1*α*1 transcription factor inducing overexpression of skeletal muscle kynurenine aminotransferase, shifting the reaction of muscle kynurenine (KYN) toward its metabolite kynurenic acid (KYNA). A major fraction of brain KYN is produced in the muscle, and if not sufficiently offsets by local metabolization, KYN is able to cross the blood-brain barrier and may cause neuroinflammation and neuronal cell death while KYNA does not. KYN may contribute to clinical depression (Agudelo et al., 2014). Aging-related increased levels of IL-1*β* and TNF-*α*, pro-inflammatory cytokines, are associated with a reduced performance on aversive memory test (Lovatel et al., 2013) underlying their role on brain networks. A two-week exercise training protocol decreased IL-1*β* and TNF-*α*, and increased histone H4 acetylation while lower levels of histone H4 acetylation were observed in hippocampi of older sedentary rats (Lovatel et al., 2013). Alternatively, a single exercise session reversed the decline in methylation of histone H3, at K9, in aged mice, further suggesting that exercise may positively influence transcriptional activity (Elsner et al., 2013) and pointing to the role of epigenetics in the communication process (*brain* ↔ *muscle com*.).

### Association with common genetic variants of hippocampus molding, regardless of training

The multi-center ENIGMA (“Enhancing Neuroimaging Genetics through Meta-Analysis”) Consortium conducted a meta-analysis of a large population sample of healthy individuals (*N* = 5775) and patients with anxiety, depression, bipolar disorder, AD or schizophrenia (*N* = 2020), computed the hippocampal volume from *3-D* anatomical T1-weighted MRI and a genome-wide association looking at the intergenic SNPs influencing the hippocampal volume normalized to intracranial volume (Stein et al., 2012; Thompson et al., 2014). Strikingly, most polymorphisms known for synaptic molding and hippocampal plasticity-modifiers showed little association in the discovery sample [*BDNF, NRG1, PICALM, TOMM40, DTNBP1, CLU, COMT*]. Only one SNP, locus rs7294919, of gene *RPL 6P15*, on chromosome 12, shows a powerful relationship with the hippocampal volume with a decrease of about 47.6 mm^3^ (Stein et al., 2012). This finding is also in line with another study (Bis et al., 2012) showing the strongest association was for rs7294919, located at 12q24, between *HRK* and *FBXW8* (Bis et al., 2012; Stein et al., 2012). *HRK* is expressed at high levels in the amygdala, entorhinal cortex and hippocampus and elsewhere in the brain. The HRK protein regulates neuronal apoptosis via a pathway involved in aging and AD (Bis et al., 2012). *FBXW8* encodes a protein involved in the ubiquitin proteasome system suggesting a role for removal of aggregating toxic protein such as the Tau protein. In another study, the hippocampal volume showed another intergenic association near the *HRK* gene (rs77956314; 12q24.22) (Hibar et al., 2015). The hippocampal volume is known to be reduced in major depression, AD or schizophrenia (Wright et al., 2000; Videbech and Ravnkilde, 2004; van de Pol et al., 2006, 2007; Jack et al., 2010). White matter projection tracts which may be identified using FA (fractional anisotropy) measurements extracted by DTI, reflecting fiber density, axonal diameter, and myelination in white matter, were found to be highly heritable (approximately 70–80% of the total phenotypic variance) (Kochunov et al., 2015).

These genome-wide associations did not evaluate any interventional inference (e.g., exercise training) and its epigenetic component. It may be hypothesized that other genes (e.g.,*BDNF*), possibly upregulated by skeletal muscle exercise, are modifying the hippocampal phenotype.

## Questions about inference of physical and mental activities

Puzzling questions arise about how factors are intertwined or independently promoting neuronal plasticity. What are the underlying mechanisms molding brain networks and enhancing fluid intelligence as well as emotional and behavioral patterns? Would mental practice alone be sufficient for synaptic remodeling? Would skeletal muscle solicitation alone cause synaptogenesis? What are the underlying mechanisms of synaptogenesis promoting the communications (*brain* ↔ *muscle com*.) and (*brain* ↔ *brain com*.) in such trainings? A recent study provides clues on how aforementioned training factors interplay in neuronal plasticity and brain network configuration (Gard et al., 2014). Gard et al. (2014) designed a case-control study comparing fluid intelligence and brain functional network architecture in three groups (Yoga or meditation practitioners, and controls) matching for demographics data. This study may suggest further insights on the respective role of mental practice and physical activity in neuronal plasticity. Association of mental practice and physical activity was reflected into slower age-related decline in fluid intelligence, a shorter normalized characteristic path length, greater small-worldness, greater global efficiency, lower clustering coefficient, greater resilience of resting state networks. Mindfulness was correlated to fluid intelligence and brain functional resilience and integration. For some measures of functional integration and segregation, the trend was slightly more statistically significant in Yogis than meditators. It is plausible that the greater network resilience in Yogis practitioners may have been explained by a longer period of past training at the time of the measurements. Indeed, meditation certainly requires only minimal motor execution and cardio-respiratory activation while Yoga solicits both mental practicing associated to actual sustained sub-maximal skeletal muscle activity. Key features from the specific training of both groups also highlights possibilities: would the combination of both mental practice and physical activity be required? A 6-month period of intermittent exercise alone (dancing), without improvement of maximal aerobic capacity (VO_2max_), suffices for cognitive improvement (Kattenstroth et al., 2013) and plausible underlying neurogenesis. However, the 6-month protocol is not improving fluid intelligence. Alternatively, would further enhancement of maximal aerobic capacity be necessary to further improve cognitive abilities at an even higher level? Mental practice is also multi-faceted as well as would its potential impact be on brain networks. Ultimately, the ancillary question should rather be: would longer and greater mental and/or physical training during one's lifetime matter more?

### Conflict of interest statement

The author declares that the research was conducted in the absence of any commercial or financial relationships that could be construed as a potential conflict of interest.
